# Assessment of medication adherence among Lebanese adult patients with non-communicable diseases during COVID-19 lockdown: a cross-sectional study

**DOI:** 10.3389/fpubh.2023.1145016

**Published:** 2023-06-21

**Authors:** Diana Malaeb, Hala Sacre, Sara Mansour, Chadia Haddad, Abir Sarray El Dine, Tamara Fleihan, Souheil Hallit, Pascale Salameh, Hassan Hosseini

**Affiliations:** ^1^College of Pharmacy, Gulf Medical University, Ajman, United Arab Emirates; ^2^School of Pharmacy, Lebanese International University, Beirut, Lebanon; ^3^INSPECT-LB (Institut National de Santé Publique, d’Épidémiologie Clinique et de Toxicologie-Liban), Beirut, Lebanon; ^4^School of Health Sciences, Modern University for Business and Science, Beirut, Lebanon; ^5^Gilbert and Rose-Marie Chagoury School of Medicine, Lebanese American University, Byblos, Lebanon; ^6^School of Medicine and Medical Sciences, Holy Spirit University of Kaslik, Jounieh, Lebanon; ^7^Applied Science Research Center, Applied Science Private University, Amman, Jordan; ^8^Department of Research, Psychiatric Hospital of the Cross, Jal Eddib, Lebanon; ^9^Faculty of Pharmacy, Lebanese University, Hadath, Lebanon; ^10^Department of Primary Care and Population Health, University of Nicosia Medical School, Nicosia, Cyprus; ^11^School of Medicine, Lebanese American University, Byblos, Lebanon; ^12^INSERM U955-E01, IMRB, Henri Mondor Hospital, Créteil, France; ^13^Department of Neurology, Henri Mondor Hospital, AP-HP, Créteil, France

**Keywords:** non-communicable, diseases, adherence, medications, Lebanon, COVID-19

## Abstract

**Background:**

Medical treatment is considered a cornerstone in non-communicable diseases (NCDs) management, lack of adherence remains the main challenge that may compromise optimal therapeutic outcome achievement.

**Purpose:**

This study aimed to evaluate treatment adherence levels and associated factors among Lebanese adult patients with non-communicable diseases.

**Materials and methods:**

A cross-sectional survey conducted during the COVID-19 lockdown imposed by the Lebanese Government (between September 2020 and January 2021) enrolled 263 adult patients through an anonymous online questionnaire to assess adherence to medications using the Lebanese Medication Adherence Scale (LMAS-14).

**Results:**

Of the total sample, 50.2% showed low adherence with a total mean adherence score of 4.41 ± 3.94. The results showed that depression (*β* = 1.351) and peptic ulcer (*β* = 1.279) were significantly associated with higher LMAS scores (lower adherence). However, age between 50 and 70 (*β* = −1.591, *p* = 0.011), practicing physical exercise (*β* = −1.397, *p* = 0.006), having kidney disease (*β* = −1.701, *p* = 0.032), and an intermediate (*β* = −1.336, *p* = 0.006) to high income (*β* = −3.207, *p* < 0.001) were significantly associated with lower LMAS scores (higher adherence).

**Conclusion:**

Our study shed light on the factors affecting medication adherence in patients with non-communicable diseases. It showed that depression and peptic ulcer were associated with lower adherence, contrary to older age, exercising, having chronic kidney disease, and a higher socioeconomic status.

## 1. Introduction

Non-communicable diseases (NCDs), also known as chronic diseases, are responsible for significant premature mortality and morbidity throughout the world. They account for more than one-half of the global disease burden with a 71% mortality rate (1, 2). The rise in the prevalence and encumbrance of NCDs has been partly linked to the increased life expectancy that resulted from the decline in premature deaths caused by communicable diseases (1).

NCDs usually last 1 year or more and require ongoing comprehensive medical care, including the assessment of daily life activities. They are a consequence of several physiologic, genetic, and behavioral factors (2), and encompass many diseases, such as cardiovascular, cerebrovascular, cancer, endocrine, and respiratory conditions (2). The development of NCDs is strongly associated with risk factors such as smoking, unhealthy diet, alcohol consumption, physical inactivity, obesity, hyperglycemia, and hyperlipidemia (3). In Lebanon, a developing middle-income country in the Middle East, NCDs are ubiquitous, with hypertension affecting one-third of the Lebanese population and dyslipidemia is around 10 times higher than in Western countries (4, 5).

Although medical treatment is considered a cornerstone in NCD management, lack of adherence remains the main challenge that may compromise optimal therapeutic outcome achievement. Adherence is the extent to which behaviors meet the agreed recommendations of a health care provider (e.g., taking medications, following a diet, or changing lifestyle) (6). It is estimated that approximately 50% of the patients diagnosed with NCD are non-adherent, leading to poor outcomes and increased morbidity and mortality (6). The underlying reasons for lack of medication adherence are complex and include lack of access to medications, cultural sensitivity, relationship with healthcare professionals, and patients’ beliefs (7). A study of Middle Eastern populations in 2011 found that non-adherence rates varied from 1.4 to 80%; the highest was among hypertensive patients (7), and the two most reported barriers were forgetfulness and drug side effects (7). In Lebanon, patients with hypertension (8), and diabetes (9) showed low treatment adherence, mainly because of forgetfulness. One of the most influential factors that can affect adherence to medications is the patient belief regarding his medications. Patient beliefs encompass individuals’ attitudes, perception, and expectations about medications. Patient beliefs and concerns about medications risks, side-effects, or long-term effects of medications are proposed to influence the degree to the adherence to the medications.

Following these results, a new scale, the Lebanese Medication Adherence Scale (LMAS-14), was developed to assess the level of medication adherence, considering Lebanese socioeconomic, psychological, occupational, economic, annoyance, and cultural factors, including forgetfulness, marital status, education level, and monthly income (10).

In Lebanon, several factors hamper drug adherence due to the combined economic, political, and sanitary crises of the past 2 years, added to the Lebanese financial collapse and the Beirut port explosion that led to severe drug shortages (11, 12).

Therefore, since medication adherence in Lebanon is facing many challenges during this period and since the burden of NCDs is continuously growing, this study aimed to assess medication adherence levels and factors that affect it among a sample of Lebanese adult patients with chronic conditions, using the Lebanese Medication Adherence Scale.

## 2. Materials and methods

### 2.1. Study design and procedure

This online cross-sectional survey, carried out between September 2020 and January 2021, enrolled 263 adult patients from all Governorates (Beirut, Mount Lebanon, North, South, and Beqaa), using the snowball sampling method because of the COVID-19 lockdown imposed by the Lebanese Government. The questionnaire was created on Google Forms and shared on social media (WhatsApp, LinkedIn, and Facebook). This study is part of a larger research project about knowledge of NCDs that included all individuals aged 18 years and above and excluded those with history of mental diseases that can compromise the ability to read and understand the questionnaire. Participation was voluntary, and anonymity was guaranteed during the data collection process.

### 2.2. Sample size calculation

According to the Epi Info software version 7.2 (population survey), the minimum sample size required to ensure a 95% confidence interval was 223 participants, based on medication adherence of 82.4% in participants with hypertension and a 5% error (10). The reason for oversampling is that this study is part of a larger research project necessitating a bigger sample size.

### 2.3. Questionnaire

The self-administered questionnaire was in Arabic, the native language in Lebanon, and required approximately 20 min to complete.

The first section of the questionnaire covered the sociodemographic and socioeconomic factors, including age, smoking status, marital status (married versus others), employment status (employed versus unemployed), family income, residence (urban versus rural), education level, past medical history (e.g., hypertension, diabetes mellitus, dyslipidemia). Age was categorized into four groups (18–29, 30–49, 50–70, and above 70 years) while family income was divided into three categories: low (<1,500,000 Lebanese Lira), intermediate (1,500,000–3,000,000 Lebanese Lira), and high (>3,000,000 Lebanese Lira) (13).

The second section consisted of the Lebanese Medication Adherence Scale-14 (LMAS-14) used to assess medication adherence. This scale covers the occupational, psychological, annoyance, and economic domains; it was previously validated among Lebanese patients with hypertension and hypothyroidism (10, 14). The dichotomous version of the LMAS-14 was used to make self-assessment simpler and less problematic, with questions rated 0 (Yes) and 1 (No), where lower scores would indicate higher adherence. The dichotomous format has some advantages, as it forces people to fall on one side of a scale or the other and is quicker to answer than questions that rely on a Likert scale, with no substantial loss of information, reliability, or validity.

### 2.4. Ethics approval

The study was conducted based on the declaration of Helsinki and was approved by the ethics committee at the School of pharmacy of the Lebanese International University (202ORC-035-LIUSOP). Written informed consent was obtained from participants before inclusion in the study.

### 2.5. Statistical analysis

Data collected were analyzed using the Statistical Package for Social Sciences (SPSS) version 25.0. Continuous variables were presented as mean and standard deviation and 95% confidence interval. Categorical and ordinal variables were shown as frequencies (n) and percentages (%). The LMAS score, taken as a continuous variable, was considered as the outcome variable, whereas sociodemographic variables and past medical diseases were considered as explanatory variables. Non-parametric tests were used as LMAS-14 was not normally distributed. The Mann–Whitney test and the Kruskal-Wallis test were used to compare the means of two groups and three groups or more, respectively. Stepwise linear regression was conducted, taking the LMAS total score as the dependent variable. Independent variables that showed a *p* < 0.2 in the bivariate analysis were entered in the final model to minimize confounding. A *p* < 0.05 was considered significant.

## 3. Results

### 3.1. Sociodemographic characteristics

Table 1 presents the sociodemographic features of the participants. The majority of the participants were females (71.5%), 18–49 years (81.4%), had a university level of education (90.5%), and a low to intermediate monthly income (90.9%). More than half of them were single (50.2%) and employed (59.3%); only 29.3% were smokers, and 30% practiced physical exercise. Of the total sample, 50.2% had low medication adherence, with a mean adherence score of 4.41 (*SD* = 3.94).

**Table 1:** Sociodemographic characteristics of the study sample.

	Frequency	Percentage
Gender		
Male	75	28.5%
Female	188	71.5%
Age		
18–29	121	46%
30–49	93	35.4%
50–70	45	17.1%
Above 70 years	4	1.5%
Living area		
Beirut	125	47.5%
Bekaa	8	3%
Mount Lebanon	63	24%
North	22	8.4%
South	45	17.1%
Marital status		
Single	132	50.2%
Married	131	49.8%
Education level		
Scholar level	25	9.5%
University level	238	90.5%
Employment status		
Unemployed	107	40.7%
Employed	156	59.3%
Income level		
Low	102	38.8%
Intermediate	137	52.1%
High	24	9.1%
Smoking status		
Yes	77	29.3%
No	186	70.7%
Exercise status		
Yes	79	30%
No	184	70%
	Mean	SD
LMAS scale	4.41	3.94

### 3.2. History of medical diseases

Table 2 presents the frequency and percentage of past medical diseases. Of the total sample, 16.9% (*n* = 92) participants had dyslipidemia, followed by 16.1% (*n* = 88) with obesity, and 15% (*n* = 82) with peptic ulcer.

**Table 2:** Description of history of medical disease.

	Count	Percentage %
Hypertension	65	12%
Diabetes	38	7%
Dyslipidemia	92	16.9%
Arrhythmias	83	15.2%
Kidney disease	23	4.2%
Peptic ulcer	82	15%
Depression	74	13.6%
Obesity	88	16.1%

### 3.3. Bivariate analysis

The results of the bivariate analysis are summarized in Table 3. A higher mean LMAS-14 score was found in females vs. males (4.89 vs. 3.20, *p* = 0.001), married vs. single participants (4.95 vs. 3.87, *p* = 0.027), those who do not practice physical exercise vs. those who do (4.76 vs. 3.58, *p* = 0.026), and those with a low monthly income vs. other groups. A higher mean LMAS-14 score was also found in participants who had arrhythmia (5.11 vs. 4.08, *p* = 0.05), peptic ulcer (5.54 vs. 3.90, *p* = 0.002), and depression (5.69 vs. 3.90, *p* = 0.003) compared to those who did not have these diseases.

**Table 3:** Bivariate analysis of the LMAS scale as the dependent variable.

	LMAS scale	*p*-value
	Mean ± SD	
Gender		
Male	3.20 ± 3.34	**0.001**
Female	4.89 ± 4.06	
Age		
18–29	4.75 ± 3.87	0.082
30–49	4.63 ± 3.98	
50–70	3.04 ± 3.66	
Above 70 years	4.00 ± 6.05	
Marital status		
Single	3.87 ± 3.62	**0.027**
Married	4.95 ± 4.17	
Income level		
Low	5.39 ± 4.27	**<0.001**
Intermediate	4.07 ± 3.68	
High	2.17 ± 2.40	
Exercise status		
Yes	3.58 ± 3.77	**0.026**
No	4.76 ± 3.96	
Hypertension		
Yes	3.80 ± 4.31	0.152
No	4.61 ± 3.79	
Diabetes		
Yes	4.21 ± 4.13	0.740
No	4.44 ± 3.91	
Cholesterol		
Yes	4.24 ± 4.15	0.613
No	4.50 ± 3.82	
Arrhythmias		
Yes	5.11 ± 3.98	**0.05**
No	4.08 ± 3.88	
Kidney disease		
Yes	3.30 ± 4.01	0.160
No	4.51 ± 3.92	
Peptic ulcer		
Yes	5.54 ± 3.97	**0.002**
No	3.90 ± 3.82	
Depression		
Yes	5.69 ± 4.60	**0.003**
No	3.90 ± 3.53	
Obesity		
Yes	4.10 ± 4.01	0.375
No	4.56 ± 3.89	

Bold values represent significant results.

### 3.4. Multivariable analysis

A first linear regression taking the LMAS-14 scale as the dependent variable and the sociodemographic characteristics as the independent variables showed that being married (*β* = 1.234, *p* = 0.011) was significantly associated with higher LMAS-14 scores (lower adherence). However, being 50–70 years old (*β* = −1.906, *p* = 0.003), practicing physical exercise (*β* = −1.343, *p* = 0.010), and having an intermediate (*β* = −1.336, *p* = 0.007) to high income (*β* = −3.331, *p* < 0.001) were significantly associated with lower LMAS-14 scores (higher adherence) (Table 4, model 1).

**Table 4:** Multivariable analysis.

Model 1: Linear regression taking the LMAS scale as the dependent variable and the sociodemographic as independent variables					
	Unstandardized beta	Standardized beta	*p*-value	Confidence interval	
				Lower bound	Upper bound
Exercise status (yes vs. no*)	−1.343	−0.156	**0.010**	−2.359	−0.327
Marital status (married vs. single*)	1.234	0.158	**0.011**	0.282	2.185
Age (50–70 vs. 18–29*)	−1.906	−0.186	**0.003**	−3.154	−0.659
High income vs. low*	−3.331	−0.247	**<0.001**	−5.016	−1.646
Intermediate income vs. low*	−1.336	−0.171	**0.007**	−2.311	−0.362

Model 2: Linear regression taking the LMAS scale as the dependent variable and the history of medical disease as independent variables					
	Unstandardized beta	Standardized beta	*p*-value	Confidence interval	
				Lower bound	Upper bound
Depression (yes vs. no*)	1.351	0.157	**0.009**	0.342	2.361
Peptic ulcer (yes vs. no*)	1.279	0.151	**0.011**	0.292	2.266
Kidney disease (yes vs. no*)	−1.701	−0.126	**0.032**	−3.257	−0.145
Exercise status (yes vs. no*)	−1.397	−0.163	**0.006**	−2.384	−0.410
Marital status (married vs. single*)	1.193	0.153	**0.012**	0.268	2.118
Age (50–70 vs. 18–29*)	−1.591	−0.155	**0.011**	−2.814	−0.367
High income vs. low*	−3.207	−0.238	**<0.001**	−4.844	−1.570
Intermediate income vs. low*	−1.336	−0.171	**0.006**	−2.286	−0.386

Variables entered: age, gender, marital status, exercise status, income level, hypertension, diabetes, cholesterol, arrhythmias, kidney disease, peptic ulcer, depression, obesity.

*Stands for reference category.

Bold values represent significant results.

A second linear regression taking the LMAS-14 scale as the dependent variable and the history of medical diseases as the independent variable showed that being married (*β* = 1.193, *p* = 0.012), having a depression (*β* = 1.351), and peptic ulcer (*β* = 1.279) were significantly associated with higher LMAS-14 scores (lower adherence). However, being 50–70 years old (*β* = −1.591, *p* = 0.011), practicing physical exercise (*β* = −1.397, *p* = 0.006), having kidney disease (*β* = −1.701, *p* = 0.032), and having an intermediate (*β* = −1.336, *p* = 0.006) to high income (*β* = −3.207, *p* < 0.001) were significantly associated with lower LMAS-14 scores (higher adherence) (Table 4, model 2).

## 4. Discussion

To our knowledge, no previous studies have explored medication adherence among Lebanese patients with NCDs, a considerable challenge in achieving therapeutic outcomes. Although poor adherence is one of the principal causes of non-responsiveness to medications, factors known to influence it have not been thoroughly investigated yet. Hence, this study assessed the medication adherence and associated factors in a sample of Lebanese adult patients with NCDs. It revealed that almost half of the patients were not adherent to their medications, highlighting that married participants, those depressed, and diagnosed with peptic ulcer had significantly lower adherence. However, participants 50–70 years old, practicing physical exercise, having a history of kidney disease, and an intermediate to high income had significantly higher adherence.

### 4.1. The extent of medication adherence

Our results showed that almost half of the patients were adherent to medications, similar to other findings among Lebanese diabetic patients (15). The low adherence rate in our study can be explained by the lack of structured healthcare systems, inappropriate medical coverages, and the low socioeconomic status, forcing patients to be reluctant to adhere to their treatment. This low adherence rate can also be due to the stressful events the Lebanese population is witnessing, whether related to the economic crisis or the COVID-19 pandemic, as described in previous literature, showing that stress decreased medication adherence (12). Furthermore, this study was conducted during the COVID-19 imposed lockdown, where chronic patients were confined at home and had limited access to healthcare services, thus decreasing adherence to medications. Another interpretation could be that this study was conducted among Lebanese patients with different and multiple NCDs, thus having lower medication adherence than those with a single disease (16).

### 4.2. Marital status and medication adherence

Our study highlighted that married individuals had lower medication adherence contrary to previous findings (17). Our findings can be explained by the fact that sometimes marriage can create conflicts and tension, differences in health beliefs or attitudes can lead to disagreements, and burden imposed on one partner to manage disease for a spouse with complex health needs can lead to caregiver burden and stress which all can impact medication adherence. Although marital satisfaction was not explored, a possible explanation for our results could be low marital satisfaction, unhappy marriage, along with illness, depression, heavy drinking, and more isolation (18, 19).

### 4.3. Diseases and medication adherence

Our results highlighted that patients with depression had poor medication adherence, consistent with the data from previous literature showing that psychiatric conditions were frequently associated with low adherence rates (20).

The reasons for the association between depressive symptoms and non-adherence to treatment might be the greater feelings of hopelessness, social isolation, withdrawal from social networks, and a possible decline in cognitive functioning that may affect memory, and reduce the energy to continue the medical treatment (21). In our study, patients with peptic ulcer disease had poor medication adherence, in agreement with data from previous studies (22). A possible explanation could be the metallic taste of medications used to eradicate *Helicobacter pylori* infection, as reported in previous research (23). Another reason could be the frequency of drug administration, the number of pills administered, and the complexity of eradication regimens, explaining the inverse relationship between the number of doses and adherence (24).

Our results revealed that patients with kidney disease had good medication adherence, consistent with previous findings (25, 26). A possible interpretation could be the availability of memory aids, assisting devices, and support and care from family members facilitating medication adherence (25, 26).

### 4.4. Socioeconomic status and medication adherence

In our study, higher medication adherence was significantly associated with intermediate-high socioeconomic class, in agreement with previous findings in other studies (27). These patients have greater provider/caregiver availability and regular medical follow-ups, enhancing medication adherence (28, 29). They also have adequate medical/prescription coverage and thus are more adherent (30).

### 4.5. Age and medication adherence

Older age was significantly associated with higher medication adherence, consistent with the literature. Older patients have increased interaction with the healthcare system (more appointments and interactions with physicians), a greater belief in the importance of chronic medication management, or a higher experience with managing medications (31).

### 4.6. Physical activity and medication adherence

Our results showed a significant association between higher medication adherence and patients who practice physical activity, in line with those of other studies (32). Patients who perform physical activity have lower depression, anxiety, and cognitive impairment, which improves medication adherence. Moreover, regular physical activity has favorable effects on reducing blood pressure, heart rate, blood glucose, lipid profile, and weight, which all decrease the risk of communicable diseases and minimize complications. Such benefits showed to increase medication compliance.

### 4.7. Limitations

Our study has some limitations. Its cross-sectional design prevents inferring the causality of the associations found. Information bias could exist since it was self-reported by the participants; thus, adherence may be overestimated, mainly because of social desirability. Dichotomizing the LMAS-14 scale might have a consequence of losing information and validity of the original scale. The LMAS-14 scale was not a sufficiently good tool to validly screen for patients with non-adherence to medications; however, it was suggested as a practical, simple, and non-expensive method. Although our sample was randomly selected across Lebanon through snowball sampling method using the social media, a selection bias might still exist as the majority of the study participants were of young age and educated which further hinders the generalizability of the results. Our study also did not consider other cultural factors that affect adherence to medication due to the nature of the study since it was conducted during COVID-19 lockdown and it was not conducted through face to face communication that impede the appropriate assessment. The cultural factors include beliefs and traditions that can shape individuals’ perceptions of health and illness, language barriers and health literacy that can impede effective communication between healthcare providers and patients, religious and spiritual beliefs that play vital role in healthcare decision-making, and family and social support. An overrepresentation of the Lebanese residing in Beirut and Mount Lebanon was also noted. Thus, more studies are mandated to assess adherence to medications among non communicable diseases outside the lock downs since our study is not a representative of adherence among all Lebanese population.

### 4.8. Clinical implications

Our findings suggest that educational programs for patients are warranted to increase awareness about the importance of medication adherence. Furthermore, the doctor-patient relationship could be improved to involve patients in their treatment, increasing the likelihood of adherence. The community pharmacist has a prominent role in patient adherence through counseling patients and educating them about drug–drug and drug-food interactions and the detrimental consequences of non-adherence.

## 5. Conclusion

Our study shed light on the factors affecting medication adherence in patients with non-communicable diseases during COVID-19 lockdown. It showed that depression and peptic ulcer were associated with lower adherence, contrary to older age, exercising, having chronic kidney disease, and a higher socioeconomic status. Further studies are necessary to confirm our results.

## Data availability statement

The raw data supporting the conclusions of this article will be made available by the authors, without undue reservation.

## Ethics statement

The study protocol was approved by the ethics committee at the Lebanese International University (2020RC-041-LIUSOP). Online consent was obtained from all participants on the first page of the questionnaire. The patients/participants provided their written informed consent to participate in this study.

## Author contributions

DM designed the study. SM, CH, DM, and TF drafted the manuscript. CH, SH, DM, and PS carried out the analysis and interpreted the results. AS contribute in data collection. DM, SH, PS, HH, and HS assisted in drafting and reviewing the manuscript. DM, PS, and HH supervised the course of the article. HS revised and edited the article for English language. All authors reviewed and approved the final version of the manuscript.

## Conflict of interest

The authors declare that the research was conducted in the absence of any commercial or financial relationships that could be construed as a potential conflict of interest.

## Publisher’s note

All claims expressed in this article are solely those of the authors and do not necessarily represent those of their affiliated organizations, or those of the publisher, the editors and the reviewers. Any product that may be evaluated in this article, or claim that may be made by its manufacturer, is not guaranteed or endorsed by the publisher.

## Abbreviations

NCDs: non-communicable diseases
LMAS-14: Lebanese Medication Adherence Scale
COVID-19: coronavirus disease-2019
